# A Randomised Cross‐Over Study to Evaluate the Physiological Effects of Internal Air Pressure Changes in Advanced Support Surface Design

**DOI:** 10.1111/iwj.70703

**Published:** 2025-06-08

**Authors:** Silvia Caggiari, Martin Toms, Rehorova Lucie, Zara Evans, Ralph Gordon, Paul Muckelt, Florence Mbithi, Davide Filingeri, Peter R. Worsley

**Affiliations:** ^1^ PressureLab, Skin Sensing Research Group, School of Health Sciences, the University of Southampton Southampton UK; ^2^ LINET spol. s r. o. Slaný Czech Republic; ^3^ ThermosenseLab, Skin Sensing Research Group, School of Health Sciences, the University of Southampton Southampton UK

**Keywords:** alternating air pressure matters, interface pressure, ischemia, pressure ulcer prevention, tissue perfusion

## Abstract

High specification mattresses periodically redistribute pressure using alternating air cells, offloading tissues. This study aimed to evaluate the effects of alternating air pressure gradients on sacral tissue physiology. This randomised cross‐over study recruited 15 healthy participants to test the three mattress settings (fast cycle, normal cycle, and slow cycle). Participants were asked to adopt supine, lateral, and high sitting (head of bed at 40°) postures, whilst transcutaneous tissue gas tensions and interface pressures at the sacrum were continuously monitored. Comparison between mattress settings and postures showed no statistical difference (*p* > 0.05) between peak pressure index values at the sacrum for each air inflation cycle speed setting. By contrast, a significantly higher sacral (*p* < 0.05) contact area was observed for high sitting. During high sitting, ischemic responses during both fast and normal air inflation cycle speed settings were recorded. During the slow air inflation cycle speed, most participants (60%–100%) showed high levels of perfusion. The present study identified a main effect of posture on interface pressure and perfusion over the sacrum. The alternating mattress speed influenced local tissue perfusion, with the greatest changes in tissue oxygenation occurring in a high‐speed setting.

**Summary**

- The speed at which internal air in mattresses alternate can influence tissue physiology.
- The effect on tissue perfusion is dependent on lying postures.
- New mattress technologies should consider intrinsic and extrinsic factors in design philosophy.

## 1 Introduction

Pressure ulcers (PUs) are localised injuries of the skin and underlying soft tissues caused by sustained pressure, or pressure in combination with shear, commonly occurring over bony prominences [1]. Several risk factors have been identified in the development of PUs, including reduced mobility, prior history of pressure ulcers, and tissue perfusion [2]. In the last few years, these risk factors have been recognised as both a Patient Safety and Quality of Care indicator for healthcare providers in both the acute and community settings [3]. The prevention of pressure ulcers represents a high priority in all healthcare institutions, and despite the increased number of interventions to improve the efficiency of preventive strategies, the incidence of PUs remains unacceptably high, with associated treatment costs estimated at £572 million annually in the UK [4].

National and international guidelines recommend frequently repositioning patients to reduce the risk of developing PUs, typically every 2–4 h [1]. In practice, this involves the periodic redistribution of pressure through postural change, which relieves previously loaded tissue areas. In individuals with impaired mobility, this process often requires the assistance of a healthcare practitioner, which can be time‐consuming and expensive [5]. Furthermore, in healthcare settings where resources are limited, strict adherence to repositioning protocols may be suboptimal, that is, in busy hospital or community settings [6, 7]. Support surface systems aim to redistribute pressure to minimise the risk of pressure ulcers. Advanced mattress and cushion devices have the capability of alternating low pressures (ALP) at the patient interface to periodically offload vulnerable skin sites. However, the extent of their benefits over foam or static hybrid systems has not been fully demonstrated [8, 9, 10, 11]. The results from these studies are confounded by the many factors which may influence pressure ulcer development [2] and the low typical incidence (~8%–12%) in different care settings [12].

Prior performance of support surfaces has been investigated using different indicators. For example, interface pressure measurements between the individual and underlying mattress have been extensively used in both lab‐based and clinical studies [13, 14], demonstrating the significant effect of postural changes on interface pressures [15]. However, interface pressure alone cannot inform location and timeline for pressure ulcer development [16]. Several recent studies examined the temporal effects of applied pressures on a range of measures indicative of the local physiological status of skin and soft tissues [17, 18, 19, 20]. These indicated that changes in local tissue perfusion, measured with transcutaneous gas tensions (T_c_PO_2_ and T_c_PCO_2_) can reflect the physiological response of skin tissues to different postures and mattress settings [21, 22]. In addition, thermodynamic conditions in the form of temperature and humidity strongly influence a person's susceptibility to PUs, leading to a growing development of microclimate management (MCM) systems at the loaded‐skin support interface [23, 24, 25].

In contrast to static mattresses, the air in alternating pressure support surfaces is channelled through a pump to cells which inflate and deflate, providing periodic offloading to tissue. Recent studies have shown that local physiology in the form of transcutaneous tissue gas tensions can be directly modulated by these internal mattress pressures [19, 26, 27]. Equivalent changes in tissue perfusion have also been observed in postural movements that can either be self‐induced or supported by carers where mobility is restricted [21]. However, the speed and magnitude of movements may influence the local tissue perfusion [28], with the former leading to ischaemia reperfusion events [29, 30].

The design of alternating pressure mattresses has been influenced by the growing focus on patient comfort. However, there is the need to assess the clinical effect of the discrete technical parameters that go into designing an alternating pressure cycle. Indeed, the optimum speed through which air is cycled within dynamic mattress systems has yet to be established, with a large range of designs and air pump settings being deployed in health and social care settings. The need for systematic mattress testing motivated the present study, which aims to assess the effects of alternating air pressure gradients within support surfaces and their effect on sacral tissue physiology.

## 2 Materials and Methods

### 2.1 Participant Trials

A convenience sample of 15 healthy participants was recruited. Research design for the study was conducted in accordance with institutional ethics approval, XXX ETHICS‐2014‐ 12703, following an adapted published protocol [20, 21]. The study design was a randomised cross over to evaluate mattress alternating cycle time and posture.

Participants were asked to lie on an alternating pressure mattress (XXXXXXXXX) whilst they adopted three distinct postures established in a random order, namely supine lying, right lateral lying, and high sitting (head of bed at 40°), for periods of 15 min each. Participants underwent two trials characterised by different mattress settings including fast cycle, normal cycle, and slow cycle speeds, performed in a random order, whilst transcutaneous gas tensions (T_c_PO_2_ and T_c_PCO_2_ measured in mmHg), interface pressures, and internal mattress pressures were continuously recorded.

### 2.2 Test Equipment

Physiological measures of T_c_PO_2_ and T_c_PCO_2_ were monitored at the sacrum using a transcutaneous gas electrode heated to 43.5°C to ensure maximum vasodilation [16]. Each electrode was attached to a monitor (TCM4 Radiometer, Denmark), recording at a frequency of 0.3 Hz. Transcutaneous monitoring has been used to assess local tissue perfusion, providing a surrogate measure for pressure‐induced local ischaemia [17, 18]. Atmospheric and zero‐point electrode calibrations were performed as per the manufacturer's recommendations. The transcutaneous tissue gas electrode was applied to the skin with a fixation ring containing an electrolyte solution at the electrode‐skin interface. A silicon pressure‐diffusing ring surrounding the electrode was applied to minimise pressure gradients caused by the electrode [19].

Interface pressure measurements were recorded using a full body pressure monitoring system (ForeSite PT, XSENSOR Technology Corporation, Canada), which incorporates 5664 pressure measuring sensor cells, with a spatial resolution of 15.9 mm, covering a sensing area of 762 × 1880 mm. Each pressure sensel operates within a range of 5–200 mmHg (0.7–26.6 kPa), with an accuracy of ± 2 mmHg and an acquisition rate of 1 Hz. A touch screen monitor, which displays real‐time images of pressure distribution, was connected to the system. The continuous pressure monitor was calibrated by the manufacturer (Xsensor, Canada) preceding the study.

The internal pressures within the mattress were measured using an Opticare Monitor and TeraTerm software (25 Hz). Mattress pressure settings were adjusted for each participant based on their weight class from a range of 10 mmHg for the lowest weight class up to 55 mmHg for the highest weight class.

### 2.3 Bed Evoked Postures

Participants were required to wear loose‐fitting clothing and adopt a series of postures on a commercial double air deck mattress replacement system (XXXXXX) located on a standard hospital bed frame (XXXXXXX).

**FIGURE 1:**
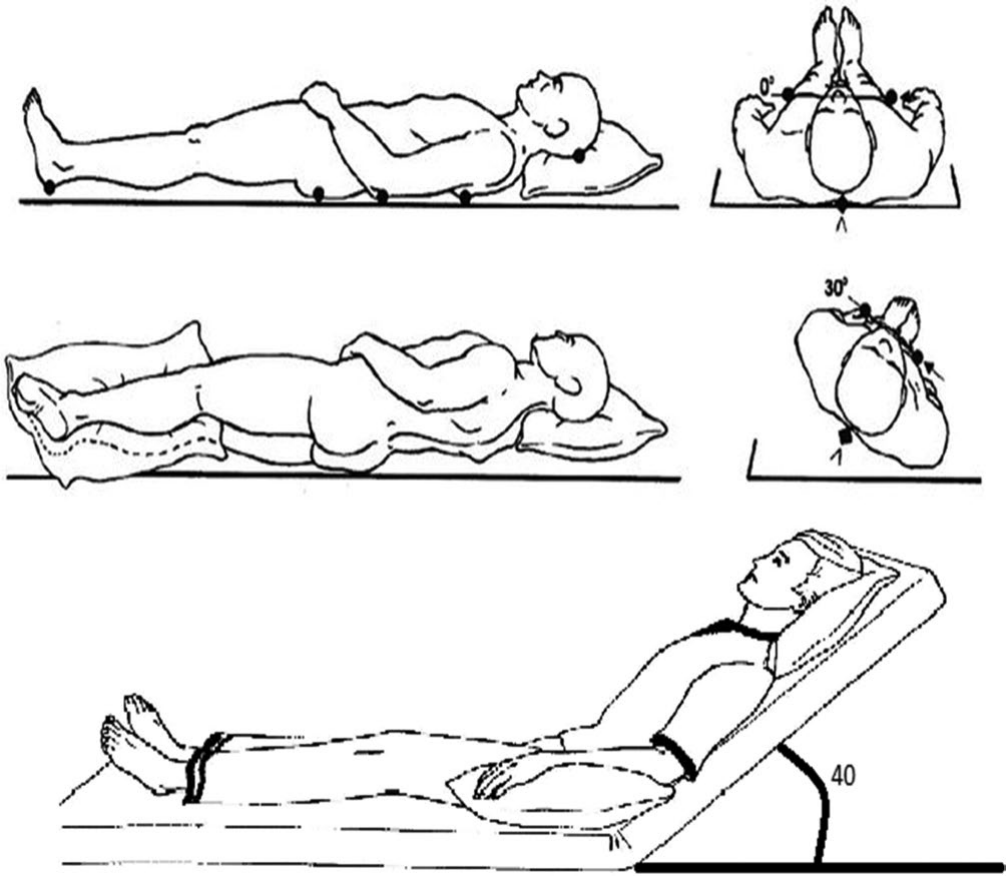
Postures adopted during testing which included supine (top), 30° tilt supported by pillows (middle) and semi‐fowler (bottom).

Prescribed sagittal and lateral changes in posture were performed, which included supine, semi‐fowler position (head of bed 40°) and a 30^o^ lateral tilt (Figure 1). When the mattress was set in the semi‐fowler position (40° head of bed), an automated boost function was initiated to increase the internal mattress pressures and prevent ‘bottoming out’. The bed frame has an autoregression feature during head of bed elevation to increase the space in the pelvic area by sliding the backrest posteriorly (110 mm).

After an acclimatisation period of 20 min for the transcutaneous tissue gas electrode to establish a baseline, each participant was asked to lie down in a supine posture on the mattress for 5 min, while the first interface pressure was recorded. Participants were then asked to adopt three different postures (supine, raised head of bed, right lateral tilt), which were performed in a random order, for a period of 15 min each. Within each posture, two cycles of air pressures (7.5 min in duration, each) were run. This was standardised for each mattress mode (slow, normal, fast inflation cycle mode) which was tested on separate days at least 48 h apart. Participants were asked to avoid strenuous activity 24 h prior to testing and no caffeine intake in the preceding 4 h. The rate of change for each mattress setting was:

1. Slow ~360 s deflation time
2. Normal ~180 s deflation time
3. Fast ~120 s deflation time

### 2.4 Outcome Measures and Data Analysis

Interface pressure, internal pressure of the air cells, and transcutaneous gas tension data were processed and analysed using a Matlab custom‐built code (MathWorks, US). Pressure parameters such as contact area (>20 mmHg), peak pressure index (PPI), and peak pressure gradient (PPG) were estimated from the interface pressure. These were subjected to filtering, according to previous studies from the authors [12, 13] and were averaged over the period of each posture (15 min). The transcutaneous gas data were normalised to baseline unloaded values, measured in the side lying position, and then categorised according to the following established characteristic responses [19].

- 1. Category 1: Perfused tissue, with normative TcPO_2_ and TcPCO_2_
- 2. Category 2: Partial ischemia, with reduced (> 25%) TcPO_2_ and normal TcPCO_2_
- 3. Category 3: Full ischemia, with reduced (> 25%) TcPO_2_ and increased (> 25%) TcPCO_2_

All data were examined for normal distribution prior to analysis using the Shapiro–Wilk test. Data for interface pressures were normally distributed and parametric statistics were employed (mean, standard deviation). Non‐parametric inferential statistics were applied to the categorical and interval data associated with the transcutaneous category responses and the comfort scores. Comparisons of posture and air inflation cycle speed of the dynamic mattress involved the non‐parametric (Chi Squared test) tests during the different postures, with the significance value set to *p* ≤ 0.05.

## 3 Results

### 3.1 Participant Demographics

A summary of the fifteen participants is provided in Table 1. These represent a relatively young cohort with a range of gender, height, weight, and BMI as well as the mattress settings they tested.

**TABLE 1:** A summary table of selected participants.

Participant	Age	Sex	Height (cm)	Weight (kg)	BMI	Air cycle settings
1	36	M	176	89.0	28.7	Normal, Fast
2	37	M	176	82.6	26.7	Normal, Slow
3	28	M	171	62.4	21.3	Fast, Normal
4	32	M	172	74.3	25.3	Fast, Slow
5	22	F	171	53.2	18.3	Normal, Fast
6	29	F	159	59.4	23.3	Fast, Slow
7	26	F	172	74.8	25.3	Slow, Normal
8	68	F	156	56.7	23.4	Slow, Fast
9	36	M	183	62.4	18.7	Normal, Slow
10	27	F	162	50.1	19.1	Normal, Slow
11	69	M	176	93.6	30.1	Fast, Normal
12	21	F	161	51.5	19.8	Normal, Fast
13	32	F	156	46.3	19.0	Normal, Slow
14	25	M	194	102.2	27.2	Normal, Fast
15	21	F	162	57.6	21.9	Fast, Slow

### 3.2 Interface Pressures

**FIGURE 2:**
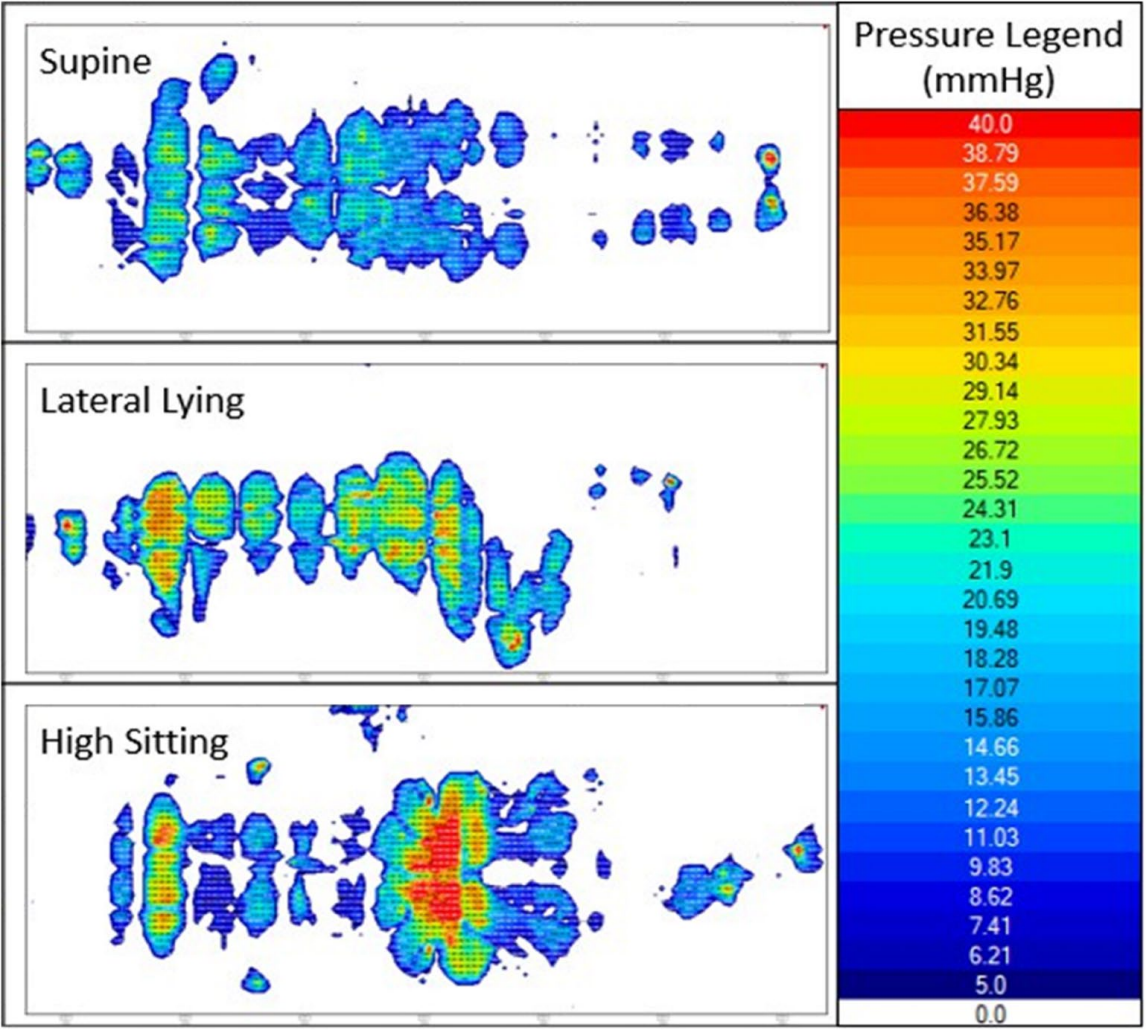
Interface pressure maps for P7 normal air inflation cycle speed in all three distinct postures: Supine, lateral lying, and high sitting with the fowler booster.

Figure 2 shows a representative trace of the spatial distribution of interface pressures. Clear changes in the contact area and point of loading can be observed when the participant adopts a supine, lateral, and high sitting posture. For example, the high sitting posture is characterised by a higher proportion of load going through the buttocks, resulting in greater pressure gradients (12.1–15.6 mmHg/cm) over the ischial tuberosities and sacral regions.

Key interface pressure parameters were exported from these pressure maps and are summarised in Table 2. Peak pressure index values varied between participants and postures, with most peak values occurring in the high sitting position. There was no statistical difference between peak pressure gradient values for each air inflation cycle speed setting across the postures. By contrast, a significantly higher value (*p* < 0.05) contact area of pressure > 20mmHg was observed for high sitting when compared to other postures in their respective mattress settings. There was a trend that fast air inflation cycle speed had the highest average pressure and slow air inflation cycle speed the lowest.

**TABLE 2:** Interface pressure values from the three postures and mattress settings.

Posture	Mattress pump setting	Mean ± StDev contact area > 20 mmHg (cm^2^)	Mean ± StDev peak pressure index (mmHg)	Mean ± StDev peak pressure gradient (mmHg/cm)
Supine	Fast	642 ± 397	42.9 ± 7.0	9.4 ± 2.3
	Normal	659 ± 487	43.0 ± 7.9	9.5 ± 2.5
	Slow	546 ± 397	41.3 ± 6.7	9.2 ± 2.0
Lateral tilt	Fast	711 ± 449	53.4 ± 36.3	10.9 ± 3.8
	Normal	721 ± 553	47.3 ± 13.1	12.8 ± 5.9
	Slow	623 ± 396	41.2 ± 9.6	9.9 ± 3.3
High sitting	Fast	850 ± 539	49.8 ± 16.9	12.1 ± 4.6
	Normal	872 ± 504	53.6 ± 21.5	15.6 ± 12.5
	Slow	781 ± 217	50.6 ± 11.8	11.7 ± 4.4

The peak pressures varied depending on mattress and posture (Table 2). There was no significant effect of posture or mattress setting on peak pressure index or pressure gradients over the sacral area, with a high degree of immersion achieved throughout.

### 3.3 Transcutaneous Gas Pressures

**FIGURE 3:**
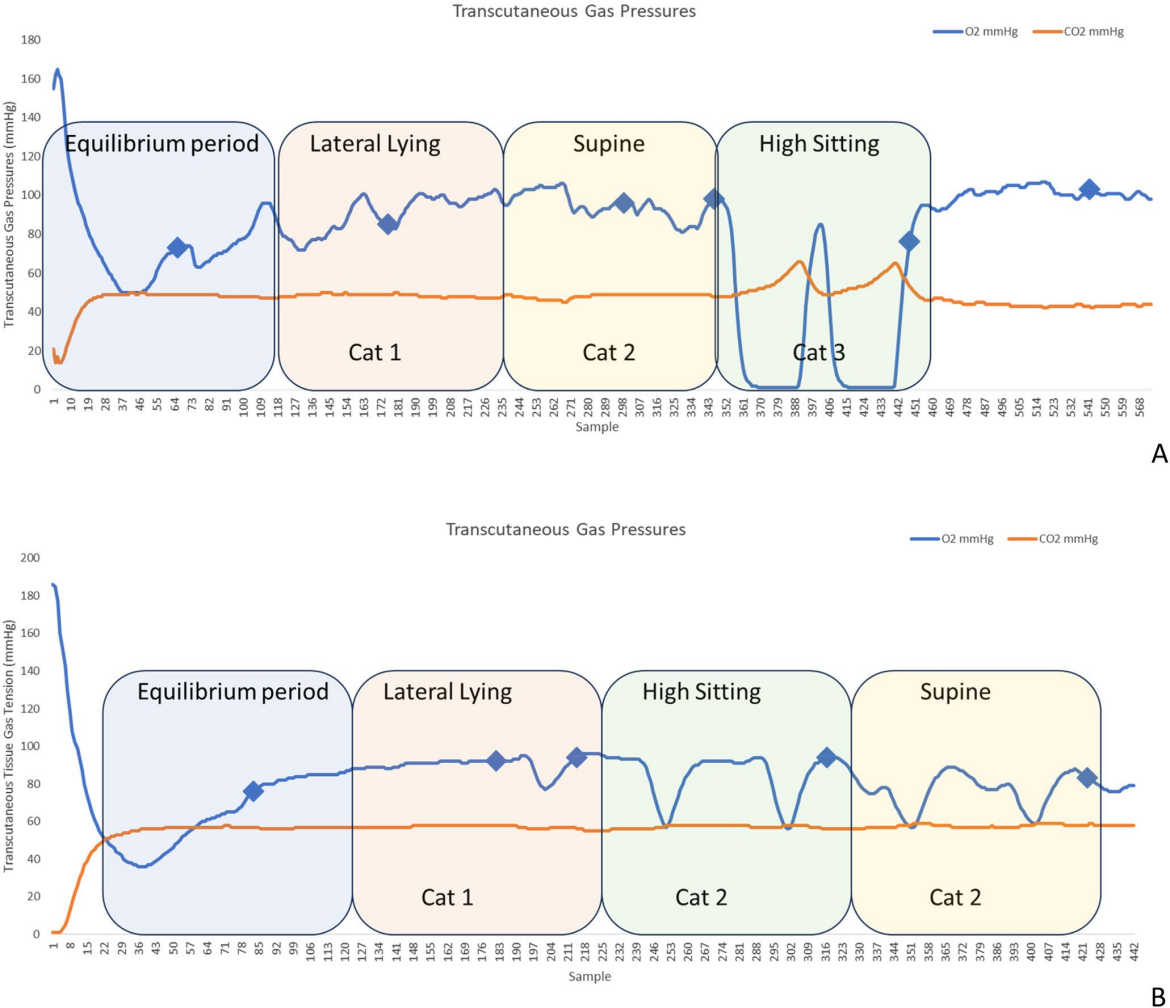
(A) P4 fast air inflation cycle speed demonstrating a category 3 tissue response during high sitting, (B) P5 Normal air inflation cycle speed demonstrating a category 2 tissue response during high sitting and supine.

Figure 3 depicts the transcutaneous oxygen and carbon dioxide values for two participants (P4 and P5) tested on different mattress settings. For P4 fast air inflation cycle speed, there were minor changes during lateral tilt and supine postures, with oxygen and low carbon dioxide values approximately similar to the baseline throughout the monitoring period. By contrast, P4 fast air inflation cycle speed during high sitting revealed a large decline (> 25%) in TcPO2 values at the sacrum and a corresponding increase in TcPCO2, which correspond to a category three response (Figure 3A). It is of note that during this posture, there was a period of recovery, associated with the deflation of an air cell over the sacral site. P5 revealed only modest changes in TcPO2 and static TcPCO2 values through the monitoring period. Where there are fluctuations in TcPO2 during supine and high‐sitting postures (Figure 3B), these are modulated by the dynamic air cell movement of the mattress system (cyclic increases and decreases).

Transcutaneous gas tension results were collated for each participant with respect to their mattress setting and posture (Table 3). The data depict that during lateral lying a high level of perfusion was observed for all participants (category 1). By contrast, during high sitting, there were three category three responses during both fast and normal air inflation cycle speed settings on the mattress. Indeed, there was a trend of an increased tissue response for trials conducted in the fast air inflation cycle speed: over half of the fast air inflation cycle speed trials revealed a category two response or higher for supine and high sitting postures. Contrastingly, for slow air inflation cycle speed, most participants had high levels of perfusion during supine, lateral, and high sitting postures. Due to the inter‐subject variability, there were no significant differences (*p* > 0.05) between mattress modes.

**TABLE 3:** Cross tabulation showing the tissue response categories for transcutaneous gas pressures as defined in Chai and Bader [18].

Mattress setting	Transcutaneous tissue response (percentage of the cohort)								
	Supine			Lateral			High sitting		
	Cat 1	Cat 2	Cat3	Cat1	Cat2	Cat3	Cat 1	Cat 2	Cat3
Fast	50	50	0	90	10	0	30	60	10
Normal	60	40	0	100	0	0	50	30	20
Slow	100	0	0	100	0	0	60	40	0

## 4 Discussion

This study used a quasi‐experimental protocol to evaluate the effects of alternating air pressure gradients and their effect on local tissue physiology in lying postures. In particular, the study addressed a gap in the current understanding regarding the rate of change of air pressures in dynamic mattress systems, and their effects on interface pressure and local skin and soft tissue physiology. The results revealed that there were similar trends across mattress settings, with high sitting causing the greatest increase in local sacral interface pressure values. By contrast, the rate of change in air cells directly influenced local sacral perfusion, with the fewest ischaemic responses observed with the slowest cycle times.

The results revealed that dynamic surface provided a high level of immersion with peak interface pressure index values ranging from 50 to 90 mmHg. These are similar to values observed in other studies assessing mattress systems [15, 20]. It is of note that interface pressure is not a direct surrogate for internal tissue strain, which is one of the mechanisms for pressure ulcer aetiology. However, transcutaneous tissue gas tension measures identified cyclical changes in local tissue perfusion, modulated by the alternating pressure of the dynamic mattress (Figure 3), indicative of cyclic strain on dermal microvascular vessels. The same phenomenon was observed in other alternating systems, which also reported the importance of air pressure differential and cycle time relating to this physiological effect [18, 19]. These changes were sensitive to the speed by which air was pumped through the mattress cells, with faster air flow causing greater changes in local tissue physiology. These findings have implications for developing new dynamic mattress systems and optimising air cell movement to promote tissue perfusion.

The present study corroborates a previous evaluation of dynamic mattress systems whereby alternating mattress modes were seen to directly influence tissue perfusion [19]. In the study by Chai et al. (2017) internal mattress pressure signatures were modified using a custom control unit to change the cycle time and magnitude of air pressure differential between adjacent air cells. They revealed that in 46% of cases at the extreme pressure amplitude of 100/0 mmHg, there was compromised local perfusion at the sacrum, evidenced by a decrease in T_c_PO_2_ levels and an increase in T_c_PCO_2_ above the normal range. By contrast, the present study saw a maximum of 20% of the cases in this ischaemic condition (category 3), typically observed in a high sitting posture. There were, however, several observations for reduced T_c_PO_2_ during the present study, particularly associated with the normal and fast air inflation cycle speed mattress settings. It is important to note that oxygen debt, as in the case of a Category 2 response, does not reflect continued compromise to tissue viability. Indeed, it has been reported that there is a threshold level of tissue oxygenation, representing a 60% reduction from unloaded basal values below which there is an accumulation of carbon dioxide within the loaded tissues [31].

Interface pressure measurements were monitored throughout the test period for each posture and mattress setting. There was no statistical difference between average pressure values for each air inflation cycle speed across the postures. By contrast, a significantly higher value (*p* < 0.05) for high sitting was observed when compared to other postures in their respective mattress settings. The values from the present study correspond to those previously reported for both dynamic and static air mattress studies [14, 18, 19, 20, 21, 32]. There were clear inter‐subject and within‐posture variations in peak pressure index values at the sacrum (Table 2). Accordingly, the authors question the common practice, particularly in the commercial literature, to describe the performance of APAM systems in terms of pressure distribution data at a single point in time. Additionally, it is tempting to relate the higher interface pressures associated with the high sitting posture to compromised tissue viability (Table 3). However, there is considerable evidence to suggest that interface pressures per se cannot reliably assess individual risk of tissue compromise [16]. When assessing factors affecting interface pressure values, there was a trend between body mass index and contact area/peaks. This was significant for contact area (*r* = 0.85, *p* < 0.01).

It is clear that this study is limited by the small number of participants, all of whom were young and healthy. Certain questions were not addressed in the present study, for example, whether the support offered by continuous low air pressure might have proven sufficient to maintain tissue viability with no additional need for alternating air pressure support. In addition, the effectiveness of different pressure configurations in the APAM system needs to be examined for bed‐bound subjects, who may be particularly susceptible to the development of pressure ulcers. It is evident that existing mattresses incorporating a single pressure profile will not accommodate the inter‐subject variability with respect to morphology, BMI, and other intrinsic factors. The present study only assessed each mattress condition and posture for a short period of time (15 min); future studies should extend this period over the course of several hours to reflect real‐world exposure to prolonged postures. It is also of note that the measurement parameters used in the present study do not reflect the internal tissue strains which play a crucial role in pressure ulcer development. To fully understand the mechanisms of tissue interaction with dynamic systems, future research should explore approaches to monitor changes in local tissue strain over time. The potential for computation modelling to supplement the understanding of dynamic surfaces has recently been proposed, where experimental data could be used to verify model predictions [33].

There is still much debate as to the comparable merits of APAM and continuous low‐pressure systems, with proponents of each in the clinical settings. The present work reaffirms that the support surfaces which incorporate alternating pressures can have a major influence on the physiological response of skin tissues. However, the pressure amplitudes within dynamic mattress systems are critical in determining these responses. Findings from the present study indicate that the speed in which air is passed through mattress cells, namely, fast to slow, generally provides sufficient pressure relief to maintain tissue viability. However, the fastest setting had the most effect on fluctuations in sacral perfusion, and the slow setting appeared to support high levels of perfusion irrespective of posture. These findings should be considered in the light of previous recommendations based on a RCT suggesting that both low pressure multi‐stage and single stage inflation and deflation APAMs are equally effective in reducing the incidence of pressure ulcers to below 6% [34].

## 5 Conclusion

This study was designed to demonstrate how the rate of change in dynamic mattress air cells could directly influence local tissue physiology in the sacrum during a range of postures in healthy individuals. It adds to the evidence that dynamic mattress systems can cause local offloading of tissues during deflating cycles and that the speed, magnitude, and duration of these dynamic events could be tailored and optimised to individuals to support tissue viability. This should be explored further in at‐risk patient cohorts where intrinsic factors such as age, comorbidities, and a history of skin damage may present enhanced susceptibility to pressure ulcers.

## Ethics Statement

Research design for the study was conducted in accordance with University of Southampton institutional ethics approval, FoHS ETHICS‐2014‐ 12 703.

## Consent

All participants gave written informed consent to participate.

## Conflicts of Interest

M.T. and L.R. are employees of Linet (Czech Republic).

## Data Availability

Data is available from request to the authors.
